# Effects of estrogen on higher-order cognitive functions in unstressed human females may depend on individual variation in dopamine baseline levels

**DOI:** 10.3389/fnins.2014.00065

**Published:** 2014-04-07

**Authors:** Lorenza S. Colzato, Bernhard Hommel

**Affiliations:** Institute for Psychological Research and Leiden Institute for Brain and Cognition, Leiden UniversityLeiden, Netherlands

**Keywords:** estrogen, cognition, dopamine, individual differences, “inverted-U”-shaped function

## Introduction

The gonadal steroid hormone estrogen (i.e., estradiol) seems to modulate higher order cognitive processes driven by dopamine (DA) such as learning, reward processing, working memory (WM), and inhibitory control (Hampson, 1990a,b; Maki et al., 2002; Caldu and Dreher, 2007; Dreher et al., 2007; Gasbarri et al., 2008; Colzato et al., 2010, 2012; Jacobs and D'Esposito, 2011). However, it is important to note that sex steroid hormones have been shown to impact several other neurotransmitter systems, including gamma-aminobutyric acid (GABA). Indeed, in healthy women the cortical GABA levels declines from the follicular phase (FP) to the mid luteal and late luteal phases (Epperson et al., 2002). In a recent review, Sinclair et al. (2014) suggested that the adolescent brain is shaped by the interaction between estrogen and glucocorticoids with a specific impact on DA neurotransmission. The focus of the present opinion article is not on glucocorticoid-estrogen interactions but on estrogen effects on higher-order cognition in unstressed human females.

High levels of estradiol are accompanied by increases in the attentional blink (Hollander et al., 2005) and in interference in the Stroop color-word task (Hatta and Nagaya, 2009), indicating reduced cognitive control. Moreover, the reactivity of the reward system is augmented in women during the midfollicular phase when estrogen is unopposed by progesterone (Dreher et al., 2007).

Previous studies have shown that gender differences in DA-modulated higher-order cognitive processes are restricted to a particular phase of the female menstrual cycle: the late FP, in which the estrogen level is high. Growing evidence suggests that the dopaminergic system seems to be particularly strongly affected by estrogen. After estrogen enters the brain, it is converted into cathecol estrogen, which has been considered to inhibit the catechol O-methyltransferase (Ball et al., 1972), an enzyme responsible for the degradation of DA in prefrontal cortex (PFC). Several studies have pointed out that the estrous cycle is related to augmentation in DA release associated with high levels of estrogen in rodents (Di Paolo et al., 1986; Becker et al., 2001; Dazzi et al., 2007; for review see Becker, 1999) and in monkeys (Czoty et al., 2009). Moreover, as pointed out by Czoty et al. (2009), receptor autoradiography studies have revealed that D2 receptor densities can raise in the presence of natural elevations in estrogen during the estrous cycle and after exogenous estrogen administration (Pazos et al., 1985; Di Paolo et al., 1988; Bazzett and Becker, 1994; Becker, 1999; see Di Paolo, 1994).

Interestingly, the ventral tier of the midbrain sends its DA projections to the dorsal and lateral parts of the PFC, while the dorsal tier sends its DA projections primarily to the ventral striatum, which projects strongly to ventrolateral and ventromedial PFC (Cools, 2006). As suggested by Miller (2000), reward information may be mediated by the dopamine-mediated innervation of PFC from a group of cells situated in the midbrain ventral tegmental area (VTA). Inhibitory control of behavior and thoughts seems to be driven by the frontal/basal-ganglia system. Finally, DA levels in PFC are related to the maintenance of WM information (Cools, 2006). Given these links between estrogen, DA, and PFC, it should not be surprising that PFC-depending functions, like inhibitory control, WM, and reward processing, are particularly affected by the menstrual cycle.

## Individual baseline levels of DA may exhibit differential sensitivity to estrogen

In some previous studies, estrogen seems to have modulated cognitive processes in opposite directions or in unreliable ways. For example, studies have found improved verbal working memory (Rosenberg and Park, 2002) and better performance on a test of implicit memory (Maki et al., 2002) when the estrogen level was high, while others found high levels of estrogen to have a negative effect on delayed matching-to-sample working memory task (Gasbarri et al., 2008). Jacobs and D'Esposito (2011) were the first to suggest that inconsistencies in the literature linking WM and estrogen (Maki et al., 2002; Rosenberg and Park, 2002; Gasbarri et al., 2008) may be explained by taking baseline levels of DA into account. Indeed, these authors showed that the direction of the effect estrogen has on WM depends on indices of baseline DA (as assessed by the genetic variability associated with the COMT Val^158^Met genotype).

We suggest that not only for WM, but for all cognitive processes related to DA, the effect of estrogen might depend on individual variation in baseline DA function, which follows an “Inverted-U”-shaped function. Indeed, neurotransmitters such as DA often relate to performance in a nonlinear fashion, with the best performance related to a medium level, while higher levels are likely to be counterproductive (Muly et al., 1998; Goldman-Rakic et al., 2000). This effect is explained by the existence of GABAergic interneurons with D1 receptors and inhibitory input to cortical pyramidal cells, which are related to cognitive performance. At moderate levels of dopamine release the function of these pyramidal cells (but not of the interneurons) is enhanced, which leads to better performance as compared to lower levels. But at high levels of dopamine release, the GABAergic inhibitory interneurons also get excited and start projecting the neurotransmitter GABA onto the pyramidal cortical cells. This provides them with inhibitory input, leading to impaired performance (Goldman-Rakic et al., 2000). Consistent with this picture, the impact of most dopaminergic agonist drugs is modulated by individual differences: increasing the dopamine level is likely to be beneficial for individuals whose level falls short of the optimal level but to impair the performance of individuals with medium (optimal) or high levels (Cools, 2006).

We speculate that different individuals may have different baseline levels of DA and may therefore exhibit differential sensitivity to the positive and negative effects of estrogen. Given that estrogen is associated with higher DA turnover rates, if estrogen affects the DA functioning in driving a particular cognitive function, we would expect a cognitive beneficial effect in the late FP (i.e., with the highest level of estrogen) for individuals with a low DA baseline level. In contrast, we would expect a cognitive detrimental effect in the late FP for individuals with an already optimal baseline level. That is, low baseline levels of DA, which are in general accompanied by poor cognitive performance, may be improved by high levels of estrogen. In contrast, high baseline levels of DA, commonly related to good cognitive performance, may be impaired by estrogen.

Colzato and colleagues showed, in two independent samples, that late FP was associated with both *less* efficient inhibitory output control (Colzato et al., 2010) and *more* efficient inhibitory input control (Colzato et al., 2012). Of course, we cannot exclude that this dissociation simply reflected the independence of input and output control (Johnston et al., 1995). However, if our idea that the effect of estrogen on all DA-driven cognitive processes depends on individual variation in baseline DA is correct, it is a real possibility that individual differences have modulated our previous findings. If so, it is reasonable to assume that our first study (Colzato et al., 2010) tapped a sample with an already optimal DA baseline level while the second (Colzato et al., 2012) happened to assess a sample with low DA baseline levels.

## Markers of DA base levels

The direct assessment of DA function in humans is only possible by means of positron emission tomography (PET) so far, which is, however, very expensive and highly invasive due to radioactive contamination and arterial blood sampling (Volkow et al., 2009). An ideal index of DA base levels, also used by Jacobs and D'Esposito (2011), is genetic variability related to levels of DA, which is nonetheless still a costly procedure.

Interestingly, DA can be found in high concentration in the amacrine and interplexiform cells of the retina (Bodis-Wollner and Tzelepi, 1998; Witkovsky, 2004). Abnormal color discrimination has been reported for several neuropsychiatric conditions underlying al dopaminergic functions, such as Parkinson's and Huntington's disease, Tourette syndrome, ADHD, and cocaine use (Paulus et al., 1993; Pieri et al., 2000; Melun et al., 2001; Tannock et al., 2006; Hulka et al., 2013). Roy et al. (2003) suggested that color vision impairment points to a central hypodopaminergic state. Very recently, color vision has been found to predict the efficiency in resolving response conflict given that both are driven by dopamine (Colzato et al., under revision). This raises the possibility that individual color discrimination performance predicts individual differences in sensitivity to the positive (i.e., enhancing) and negative (i.e., unfavorable) effects of estrogen. For example in tasks assessing cognitive control and adaptation one would expect benefits (e.g., better goal regulation in the face of response conflict) in the late FP for individuals with poor color discrimination but a detrimental effect (e.g., poorer goal regulation) in individuals with optimal color discrimination.

Another interesting measure of DA functioning is the spontaneous eyeblink rate (EBR), a well-established clinical indicator (Karson, 1983; Shukla, 1985; Blin et al., 1990; Taylor et al., 1999). Patients with DA-related dysfunction show atypical patterns: EBRs are elevated in in schizophrenia patients (Freed, 1980) but reduced in recreational cocaine users (Colzato et al., 2008b) and Parkinson's patients (Deuschel and Goddemeier, 1998). Moreover, pharmacological studies in nonhuman primates and humans have shown that DA agonists, such as apomorphine, and antagonists increase and decrease EBRs, respectively, (Blin et al., 1990; Kleven and Koek, 1996). Similarly to color vision, EBR has also been found to predict DA-driven cognitive processes (e.g., Dreisbach et al., 2005; Colzato et al., 2007, 2008a, 2009). Accordingly, EBR, in interaction with the individual genetic setup, should predict individual differences in sensitivity to the positive and negative effects of estrogen. In particular, we would expect cognitive benefits in the late FP for individuals with low EBR but impairments in individuals with an average/high EBR.

It might be particularly informative to use proton magnetic resonance spectroscopy (1H-MRS) and plasma levels of homovanillic acid (HVA) to trace the impact of estrogen on the DA system. 1H-MRS permits to measure the concentration of particular chemicals, based on subtle differences in the resonance of the protons they contain. This technique has been successfully applied in the past to reflect changes in dopamine pathways (Moore et al., 2006) and to investigate the effect of dopaminergic treatment on the cortex (Lucetti et al., 2007). In contrast to PET, 1H-MRS does not use invasive radioactive tracers and it is way less expensive. 1H-MRS allows measuring brain metabolites including creatine (Cr), inositol (Ino), and glutamate and glutamine (Glx), and the ratio between them. Because protons experience different shielding effects from the surrounding electrons in different molecules, their resonance varies from one type of molecule to another. In the late FP we would expect increased brain DA and, accordingly, a modulation of the Glx-to-Cr and Glx-to-Ino ratio.

Homovanillic acid (HVA) is a metabolite of DA which is typically decreased in repetitive behavior disorders (Lewis et al., 1996) and it may be altered in in disorders of catecholamine metabolism. For example, monamine oxidase-A deficiency can cause decreased HVA values, while a deficiency of dopamine beta-hydrolase (the enzyme that converts dopamine to norepinephrine) can cause elevated HVA concentrations. Accordingly, HVA values should predict individual differences in sensitivity to the positive and negative effects of estrogen. In particular, we would expect cognitive benefits in the late FP for individuals with low HVA values but impairments in individuals with an average/high HVA values.

## Summary

We propose that future studies investigating the effect of estrogen on DA-driven higher order cognitive processes should take into account individual differences in DA base levels. The existing research on the role of estrogen in higher order cognitive processes has been mainly “effect”-driven, and thus only shown *that* estrogen can have an effect without explaining *how* it modulates cognitive processes and why some people benefit more than others. To get a better understanding of the underlying mechanism and the interplay between estrogen, dopaminergic supply, and cognitive functioning it is mandatory to develop a comprehensive, detailed model of how estrogen modulates higher order cognitive processes in healthy humans.

### Conflict of interest statement

The authors declare that the research was conducted in the absence of any commercial or financial relationships that could be construed as a potential conflict of interest.
